# Research on the anti-aging mechanisms of *Panax ginseng* extract in mice: a gut microbiome and metabolomics approach

**DOI:** 10.3389/fphar.2024.1415844

**Published:** 2024-06-20

**Authors:** Longfei Lin, Ruying Tang, Yuling Liu, Zhiyong Li, Hui Li, Hongjun Yang

**Affiliations:** ^1^ Institute of Chinese Materia Medica, China Academy of Chinese Medical Sciences, Beijing, China; ^2^ Institute of Traditional Chinese Medicine Health Industry, China Academy of Chinese Medical Sciences, Nanchang, China; ^3^ China Academy of Chinese Medical Sciences, Beijing, China

**Keywords:** natural aging, ginseng, microbiota–gut–brain axis, fecal microbiota transplantation, fecal flora sequencing, metabolomics

## Abstract

**Introduction:** Aged-related brain damage and gut microbiome disruption are common. Research affirms that modulating the microbiota-gut-brain axis can help reduce age-related brain damage.

**Methods:** Ginseng, esteemed in traditional Chinese medicine, is recognized for its anti-aging capabilities. However, previous Ginseng anti-aging studies have largely focused on diseased animal models. To this end, efforts were hereby made to explore the potential neuroprotective effects of fecal microbiota transplantation (FMT) from Ginseng-supplemented aged mice to those pre-treated with antibiotics.

**Results:** As a result, FMT with specific modifications in natural aging mice improved animal weight gain, extended the telomere length, anti-oxidative stress in brain tissue, regulated the serum levels of cytokine, and balanced the proportion of Treg cells. Besides, FMT increased the abundance of beneficial bacteria of *Lachnospiraceae*, *Dubosiella*, *Bacteroides*, etc. and decreased the levels of potential pathogenic bacteria of *Helicobacter* and *Lachnoclostridium* in the fecal samples of natural aged mice. This revealed that FMT remarkably reshaped gut microbiome. Additionally, FMT-treated aged mice showed increased levels of metabolites of Ursolic acid, β-carotene, S-Adenosylmethionine, Spermidine, Guanosine, Celecoxib, Linoleic acid, etc., which were significantly positively correlated with critical beneficial bacteria above. Additionally, these identified critical microbiota and metabolites were mainly enriched in the pathways of Amino acid metabolism, Lipid metabolism, Nucleotide metabolism, etc. Furthermore, FMT downregulated p53/p21/Rb signaling and upregulated p16/p14, ATM/synapsin I/synaptophysin/PSD95, CREB/ERK/AKT signaling in brain damage following natural aging.

**Discussion:** Overall, the study demonstrates that reprogramming of gut microbiota by FMT impedes brain damage in the natural aging process, possibly through the regulation of microbiota-gut-brain axis.

## 1 Introduction

Population aging has become a growing global concern, causing anxiety among individuals and imposing a heavier burden on society. Forecasts suggest that within the next 30 years, the elderly population will hit 1.5 billion. This surge is expected to degrade the quality of life of the elderly and significantly amplify the financial burden of healthcare systems across the globe (Fang et al., 2015). Natural aging is an inevitable part of life that typically results in a gradual deterioration of molecular, cellular, and tissue functions, including those of vital organs like the gastrointestinal tract and the brain. This decrease can lead to comprehensive damage at the organism level, resulting in frailty and, ultimately, mortality. Previous research has shown that while aging is systemic, the central nervous system of the brain is most susceptible to aging, which is related to autophagy, DNA damage, and oxidative stress (Hajam et al., 2022). In addition, the brain is highly vulnerable to oxidative damage due to its high oxygen consumption and the presence of unsaturated lipids, coupled with its relatively limited antioxidant defense mechanisms (Giudetti et al., 2018). Brain aging is a major risk factor for most neurodegenerative diseases, including Alzheimer’s disease and Parkinson’s disease (Ionescu-Tucker and Cotman, 2021). Alzheimer’s disease is the most common neurodegenerative disease worldwide, and its incidence increases with age. In brief, brain aging is one of the most typical manifestations of the natural aging process. Moreover, brain aging is mainly manifested as a cognitive dysfunction, and it becomes more pronounced with age (Isaev et al., 2019). Research has shown that the occurrence of cognitive dysfunction is closely related to functional changes in the hippocampus (Culig et al., 2022). Therefore, maintaining the normal physiological function of the hippocampus can effectively alleviate the cognitive dysfunction caused by aging, thereby delaying brain aging.

The gut is one of the core organs playing an important role in digestion and absorption in the human body. Loss of appetite and intestinal dysfunction caused by intestinal flora disorder in the elderly have been confirmed to be closely related to aging (Ling et al., 2022). Age-related changes in the gut microbiota composition are closely correlated with impaired intestinal epithelial barrier function, immune function disorders, system inflammation, etc. (Molinero et al., 2023). Nowadays, an increasing number of studies focus on brain aging and gastrointestinal function decrease, with the aim of discovering potential interventions to slow down the aging process. Notably, the field of research on the microbiota–gut–brain axis is attracting significant interest, aiding researchers in unraveling the interactions and underlying mechanisms between the brain and the gut. For example, research has indicated that fecal microbiota transplantation (FMT) can increase the amount of fecal short-chain fatty acids (SCFAs) regulating the intestinal permeability on neurological restoration in a spinal cord-injury mouse model via the microbiota–gut–brain axis (Jing et al., 2021). The main participants of the microbiota–gut–brain axis include the gut microbiota, gut metabolites, and the nervous system (Hou et al., 2023). Currently, microbiota-targeted techniques (e.g., FMT), microbiota sequencing, and metabolomics offer essential technical support for research. These tools are instrumental in enhancing public comprehension of the relationship of the microbiota–gut–brain axis (Liu et al., 2022). Therefore, exploring the microbiota–gut–brain axis sheds light on the connection between brain and gut aging, guiding the development of interventions that can slow down aging and prevent age-related diseases.

Proper diet and nutrition management, such as dietary intake of theaflavins, neoagarotetraose, etc., have been shown to be capable of confronting age-related diseases and prolonging lifespan (Li et al., 2023; Li et al., 2023). Thus, a healthy diet is a safe and effective option to alleviate aging. Ginseng is a traditional Chinese medicine (TCM) with anti-aging effects due to its active components, including saponins, polysaccharides, and active peptides (Su et al., 2023). Previous research has indicated that ginseng is endowed with antioxidant, antiapoptotic, neuroprotective, and age-delaying effects (Xu et al., 2023). Ginseng as a TCM is renowned for its life-extending properties and its protective effects on various organ systems, including the cardiovascular, nervous, immune, and skin systems. It also has potent antioxidant and anti-inflammatory capabilities (Yang et al., 2017). Research has indicated that ginsenosides, ginseng polysaccharide, ginseng oligopeptide, and ginseng volatile oil exhibit significant anti-aging effects in aging mouse models (Luo et al., 2020; Yang et al., 2021; Choi et al., 2022; Su et al., 2023; Sun et al., 2023). In particular, ginsenosides Rg1, Rg3, Rb1, Rb2, and Re in ginseng water extracts are known to possess notable anti-aging effects, largely due to their influence on the gut microbiota (Huang et al., 2023; Shi et al., 2024). Therefore, ginseng is considered a non-negligible natural remedy for healthy aging and attracts extensive attention from scholars. Research has indicated that ginseng exerts anti-aging effects by regulating oxidative stress, apoptosis, inflammation, and intestinal flora on D-galactose-induced aging mice (Hao et al., 2022). Furthermore, a clinic trail has suggested that ginseng may exert an anti-aging effect by affecting the sphingolipid metabolism pathway among healthy people of different ages (Yang et al., 2021). However, previous research on the anti-aging properties of ginseng has predominantly focused on disease-induced mouse models. There is a scarcity of studies that utilize naturally aging mice for their research. Furthermore, there are no studies that use FMT to delve into the underlying mechanisms of the anti-aging effects of ginseng. In addition, the mechanisms of dietary intake of ginseng in impeding brain damage during natural aging have not been well clarified. While the anti-aging effect and mechanism of ginseng have been partially reported, the anti-aging mechanism remains unclear from the aspect of intestinal flora by transplanting fecal flora from oral ginseng mouse donors to naturally aged mice. To this end, the present study was conducted to uncover how ginseng mitigated brain aging in mice by examining the microbiota–gut–brain axis using FMT, microbiota sequencing, and metabolomics. Overall, the study bolsters the experimental proof of the anti-aging capabilities of ginseng and suggests its potential as a dietary intervention to enhance the quality of life of the elderly. Moreover, further clinical trials on the anti-aging effects of ginseng are needed to explore its benefits in humans.

## 2 Materials and methods

### 2.1 Animals

Here, C57BL/6N aged male mice (20-month-old, 31–36 g; certificate no. SCXK [32] 2016–0002) were obtained from SPF (Beijing) Biotechnology Co., Ltd. The animals were housed in a standard animal room (room temperature, 20°C–24°C; relative humidity, 30%–40%; and light condition, 12-h dark/light cycle) with free access to food and water. All the mice were fed SPF maintenance feed provided by Beijing Keao Liqi Feed Co., Ltd. All animal experiments were conducted in strict accordance with the rules of animal care and use of the Institute of Chinese Materia Medica, China Academy of Chinese Medical Sciences (IACUC Issue No. MD 2023–01–10–01).

### 2.2 Preparation and measurement of the *Panax ginseng* extract

The ginseng herbal material was sourced from Anguo Changda Chinese Medicinal Herbs Co., Ltd (Baoding, China) and authenticated by Professor Hui Li (Institute of Chinese Materia Medica, China Academy of Chinese Medical Sciences, Beijing, China) as the dried roots and rhizomes of *Panax ginseng* C. A. Mey., plants of the Acanthopanax family (hereinafter referred to as ginseng). The ginseng extract used in this experiment was prepared using the following method: 200 g of ginseng root was added to 1,600 mL water and boiled for 1.5 h. The water extract was then drained out. Subsequently, 1,600 mL water was added and boiled for 1.5 h, after which the remaining water extract was drained out again. The water extract was pooled together and concentrated to a constant value using rotary evaporators (Tokyo Rikakikai Co., Ltd. Type: N-12108V-WB). Then, the resulting concentrated liquid was frozen at −30 °C for 20 min, freeze-dried into solid using a freeze-dryer machine (Foring Science Instrument Technology Development Co., Ltd, Beijing, China), and crushed into powder for use. Finally, 68 g ginseng extract powder was obtained (the mass ratio between raw ginseng and ginseng extract powder was 200:68). During processing, 8.50 g ginseng extract powder was dissolved in 100 mL pure water, and the gavage volume for mice was 0.1 mL/10 g body weight. In addition, the details of three different fingerprinting methods, namely, the pharmacopoeia method, the methanol method, and the 70% ethanol method, and the measurement results of the ginseng extract powder in this study are provided in Supplementary Material Data S1.

### 2.3 Experimental groups and diets

**FIGURE 1:**
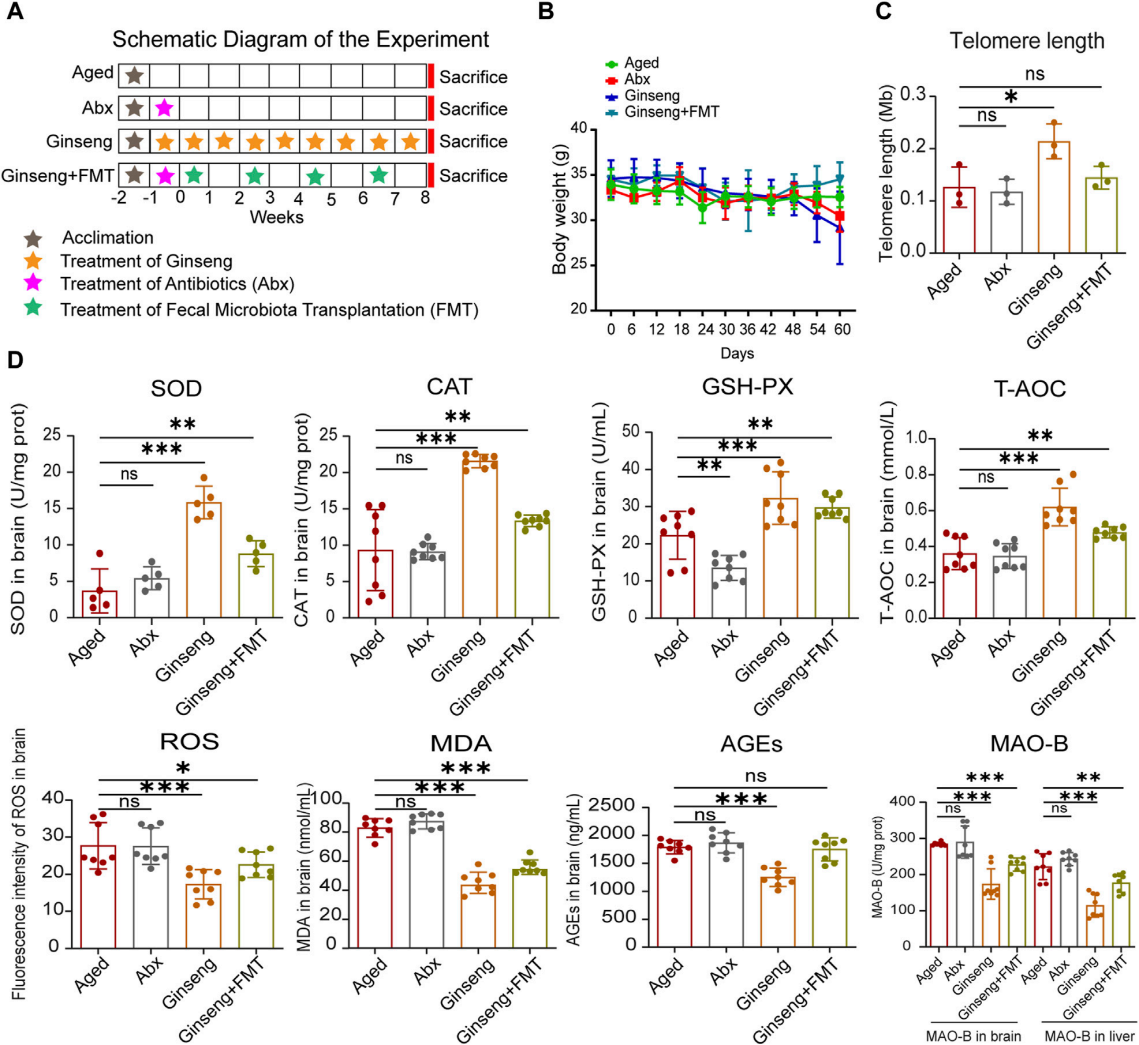
Treatment with ginseng and FMT mitigated age-associated oxidative stress in mice undergoing natural aging. **(A)** Workflow diagram of this study. The 20-month-old aged mice were grouped into four groups randomly: the aged group (aged), aged + abx group (Abx), aged + ginseng group (ginseng), and aged + ginseng + FMT group (ginseng + FMT). The fecal transplant materials were collected from ginseng group mouse donors. After a week of acclimation of all mice, ginseng group mice were given ginseng extract continuously for 9 weeks, Abx and ginseng + FMT group mice were given antibiotic treatment for a week, and then FMT was repeated four times for 4 weeks in the ginseng + FMT group only (once/week), while the aged group mice were fed water normally. **(B)** Body weight in different groups. The mice were housed in an SPF facility and weighed once a week during this experiment. Data are shown as the mean ± standard deviation (SD), n = 8 per group. **(C)** Telomere length in the brain tissues of different groups. Data are shown as the mean ± SD, n = 3 per group. Significance was determined by one-way ANOVA, followed by the LSD *post hoc* test or Dunnett’s T3 test for the comparison of multiple groups; ^***^ *p <* 0.001. **(D)** Changes in oxidative stress indexes in the brain between different groups. These oxidative stress indexes include SOD, CAT, GSH-PX, T-AOC, ROS, MDA, AGEs, and MAO-B. Data are shown as the mean ± SD, n = 8 per group. Significance was determined by one-way ANOVA, followed by the LSD *post hoc* test or Dunnett’s T3 test for the comparison of multiple groups; ^*^ *p <* 0.05, ^**^ *p <* 0.01, and ^***^ *p <* 0.001.

After 7 days of adaption, 32 C57BL/6N aged male mice were randomly divided into the aged mice group (aged), the aged mice treated with ginseng extract group (ginseng), the aged mice treated with a mixture of antibiotics group (Abx), and the aged mice treated with fecal microbiota transplantation group (ginseng + FMT). There were eight mice per group. Four mice in each group were raised in the same cage. To deplete the gastrointestinal microbiota of the mice in the Abx and ginseng + FMT groups, vancomycin (0.5 mg/mL/day; Sigma-Aldrich, United States), neomycin (1 mg/mL/day; Macklin, China), metronidazole (1 mg/mL/day; Solarbio, China), and ampicillin (1 mg/mL/day; Solarbio, China) were mixed in drinking water and given to the mice *ad libitum* for 7 days (10 mL/kg/day) (Jin et al., 2023). This antibiotic treatment was defined as the 0 week. During the 7-day period, the aged group mice were provided water as their normal diet, whereas the ginseng group mice received ginseng extract powder orally once daily. This regimen was maintained for a duration of 9 weeks, starting 1 week prior to the experiment (week −1) and continuing from week 1 through week 8 until the conclusion of the study. The daily dose of the crude drug of ginseng for human adults was 10–30 g/kg, and the middle dose of 15 g/kg for human adults was adopted in this study (Bessell et al., 2020; Hwang et al., 2020; Yang et al., 2021). Moreover, the equivalent dose in the mice was 10-fold that of adults, common in “pharmacological experimental methodology” (Guo et al., 2021), which was calculated to be 2.5 g/kg/d of ginseng raw medicine for mice (Guo et al., 2024). Briefly, the ginseng group mice were supplied with ginseng extract powder at a dosage of 0.85 g/kg/d orally in the present study. Upon the antibiotic treatment, a total of 100 μL of the resuspended fecal transplant material was given by oral gavage to the ginseng + FMT group mice daily during the first, third, fifth, and seventh weeks, while they were treated with water in the second, fourth, sixth, and eighth weeks. The ginseng group mice, which were not treated with antibiotics, served as donors for the collection of the gut microbiota under SPF conditions. This collection took place on the day of the transplant, within 2 h prior to the gavage administration, as previously described in the literature. Fresh mouse feces were collected in a sterile centrifuge tube. Then, the fresh 100 mg stools were resuspended in 1 mL of sterile saline (100 mg: 1 mL), and the supernatant was collected and used as the transplant material after mix sterile saline centrifugation at 800 *g* for 3 min (Jing et al., 2021). The workflow diagram is shown in Figure 1 A. In addition, all the mice were weighed once a week during this experiment. Finally, on the final day of the eighth week, mice from all groups were fasted for 16 h but allowed free access to water before being euthanized. Upon the conclusion of the experiment, all mice were completely anesthetized. Orbital blood samples were collected. Subsequently, plasma and serum were separated from blood samples, and the serum was then stored at −80°C. Additionally, hippocampal and liver tissues were harvested from mice across all groups and stored at −80°C. Moreover, the bone marrow of mice in each group was used for bone mesenchymal stem cells (BMSCS) isolation and flow cytometry analysis (three mice per group).

### 2.4 Measurement of the telomere length in the brain

Quantitative real-time PCR (qPCR) was adopted to measure the telomere length in the brain tissue of mice in each group according to previous research (Lai et al., 2018). The collected brain tissue was ground into fine powder in liquid nitrogen, and tissue lysate solution and proteinase K solution were then added to completely digest the tissue to extract the DNA. The concentration of the extracted genomic DNA was adjusted. Furthermore, the reaction system of the reference mouse genomic DNA samples included the telomere primer set and single-copy reference primer set, 2X GoldNStart TaqGreen qPCR master mix, and sterile aqueous solution. Subsequently, the qPCR program was performed using a multicolor real-time PCR detection system (Bio-Rad Laboratories Inc., California, Hercules, United States). The telomere length was detected using the qPCR program. Finally, the telomere length in the brain tissue of mice was calculated according to the 2-∆∆Cq formula.

### 2.5 Measurement of the ROS level in the brain

The reactive oxygen species (ROS) levels in the hippocampus of different groups were tested according to previous research (Wu J. et al., 2020). Briefly, an appropriate amount of hippocampus tissues was cleaned with PBS (4°C precooling), and then tissue digestion was performed using 0.25% trypsin. Upon centrifugation at 567 *g* for 5 min, single-cell precipitation was collected to detect the ROS levels according to the kit following the manufacturer’s instructions (E004-1-1, Nanjing Jiancheng Bioengineering Institute, China). Then, the cell precipitation was resuspended and centrifuged at 567 *g* for 5 min, the supernatant was discarded, and the cell precipitation was washed twice and resuspended again. Finally, the fluorescence intensity was detected using a fluorescence microplate (SpectraMax M5, Molecular Devices, United States) with an excitation wavelength of 488 nm and an emission wavelength of 525 nm.

### 2.6 Detection of inflammatory and oxidative stress cytokine levels in the brain and liver

For serum analysis, the blood samples were centrifuged at 3,500 *g* for 10 min at 4°C to separate the serum. The levels of serum interleukin-6 (IL-6; H007-1-2, Nanjing Jiancheng Bioengineering Institute, China), serum interleukin-2 (IL-2; H003-1-2, Nanjing Jiancheng Bioengineering Institute, China), and serum tumor necrosis factor-α (TNF-α; H052-1-2, Nanjing Jiancheng Bioengineering Institute, China) were detected using ELISA kits following the manufacturer’s instructions. In addition, the levels of growth differentiation factor 11 (GDF11; H642-1-2, Nanjing Jiancheng Bioengineering Institute, China) in the plasma of mice in each group were also identified using the ELISA kits. Furthermore, the levels of oxidative stress biomarkers in the serum were separately assessed using corresponding commercial kits, including brain superoxide dismutase (SOD; A001-3-2, Nanjing Jiancheng Bioengineering Institute, China), brain catalase (CAT; A007-1-1, Nanjing Jiancheng Bioengineering Institute, China), brain glutathione peroxidase (GSH-PX; A005-1-2, Nanjing Jiancheng Bioengineering Institute, China), brain total antioxidant capacity (T-AOC; A015-2-1, Nanjing Jiancheng Bioengineering Institute, China), brain malondialdehyde (MDA; A003-1-2, Nanjing Jiancheng Bioengineering Institute, China), brain advanced glycation end products (AGEs; CEB353Ge, Wuhan Youersheng Technology Co., Ltd., China), and brain and liver monoamine oxidase B (MAO-B; A034-1-1, Nanjing Jiancheng Bioengineering Institute, China).

### 2.7 Flow cytometry analysis of immune cells in the brain

As previously described (Luo et al., 2019), single-cell suspensions of mouse spleen tissue and peripheral blood were prepared. Subsequently, CD25 and CD4 antibodies were added to the single-cell suspensions using the True-Nuclear™ Mouse Treg Flow™ Kit (FOXP3 Alexa Fluor^®^ 488/CD4 APC/CD25 PE, 320029, BioLegend), and the blank control group was set up (without any antibody). Then, the cells were incubated at 4 C in the dark for 30 min. The samples were centrifuged at 500 *g* for 5 min to pellet the cells. The supernatant was discarded, and the cell precipitate was resuspended. This centrifugation and resuspension process was repeated to achieve a consistent cell suspension. Transcription Factor Fixation/Permeabilization Buffer was added to the cell suspension and incubated for 30 min at 4 C in the dark. The resuspended cells were treated with a permeabilization buffer, followed by the addition of FOXP3 antibodies to label the cells. Finally, the cells were washed twice, resuspended in flow loading buffer, and tested. All samples were analyzed using a FACSCanto II cell analyzer (BD Biosciences, NJ, United States). The acquired data were analyzed utilizing FlowJo software (FlowJo 10.8.1). The following antibodies were used: mouse anti-mouse CD3/PE antibody (bsm-30149A-PE, Bioss), mouse anti-mouse CD4/APC antibody (BF40033, Bioss), and FITC anti-mouse CD8a antibody (100804, BioLegend).

### 2.8 Western blotting

Hippocampal tissues were homogenized using liquid nitrogen, and a lysis liquid for bone marrow mesenchymal stem cells was prepared through ultrasonication. Tissue Extraction Reagent II (Thermo Fisher Scientific) with a protease inhibitor (Roche, Switzerland) and a phosphatase inhibitor (Roche, Switzerland) was used to extract DNA. Upon centrifugation at 13,400 *g* at 4°C for 15 min, homogenates of hippocampal tissue were obtained from the supernatant. Subsequently, the protein concentration was determined by BCA protein quantification. Based on the quantification results, the protein loading buffer was adjusted to a concentration of 5 mg/mL. Then, 30 μg of protein lysates were size-separated using 10% SDS-PAGE, transferred to PVDF membranes with a diameter of 0.22 mm (Pall), blocked with 5% skim milk (Coolaber, China) for 1 h at room temperature, and incubated in the primary antibody overnight at 4°C and the secondary antibody for 1 h at room temperature. Finally, visualization was achieved using the ECL chemiluminescence reagent (Tanon, China). The primary antibody included p53 mouse monoclonal antibody (BF8013, Affinity), p21 Cip1 antibody(AF6290, Affinity), CDKN2A/p16INK4a antibody (AF5484, Affinity), ATM antibody (AF4119, Affinity), retinoblastoma antibody (AF6103, Affinity), synapsin I antibody (AF6201, Affinity), synaptophysin antibody (AF0257, Affinity), PSD95 antibody (AF5283, Affinity), CREB mouse monoclonal antibody (BF8028, Affinity), phospho-CREB (Ser133) antibody (AF3189, Affinity), ERK1/2 antibody (AF0155, Affinity), pan-AKT1/2/3 antibody (AF6261, Affinity), phospho-ERK1/2 (Thr202/Tyr204) antibody (AF1015, Affinity), and phospho-AKT1/2/3 (Ser473) antibody (AF0016, Affinity).

### 2.9 Detection of fecal microbiota, 16S rRNA sequencing, and bioinformatics analysis

As previously described (Wu et al., 2020), DNA from the fecal samples was extracted according to the manufacturer’s instructions using the DNeasy PowerSoil Pro Kit (QIAGEN, Inc., Netherlands). Genomic DNA was extracted from the feces using sodium dodecyl sulfate, and the purity and concentration of DNA were detected using agarose 1% gel electrophoresis. An appropriate amount of genomic DNA was taken and diluted to 1 ng/μL in sterile water in a centrifuge tube. The template-diluted DNA sample (1 ng/μL) and Phusion^®^ High-fidelity PCR Master Mix with GC Buffer (New England Biolabs) were used to amplify the 16S rRNA V4 gene marker. Each DNA sample of the bacterial 16S rRNA gene was amplified with primers 515F (5′-GTTTCGGTGCCAGCMGCCGCGGTAA-3′) and 806R (5′-CAGATCGGACTACHVGGGTWTCTAAT-3′). The amplicons obtained by PCR were analyzed by 2% agarose gel electrophoresis, and a band of a desired size was purified using the QIAquick Gel Extraction Kit (QIAGEN, Inc., Netherlands). The TruSeq^®^ DNA PCR-Free Sample Preparation Kit was used for library construction. The qualified library was then sequenced on the second-generation NovaSeq 6000 platform. In addition, microbiome bioinformatics was performed using QIIME 2 2019.4 (Bolyen et al., 2018) with slight modifications according to the official tutorials (https://docs.qiime2.org/2019.4/tutorials/). Microbiome bioinformatics analysis was mainly performed using QIIME 2 2019.4 (Bolyen et al., 2018), while the OTU clustering procedure was conducted following the VSEARCH (v2.13.4) (Rognes et al., 2016) pipeline (https://github.com/torognes/vsearch/wiki/VSEARCH-pipeline).

### 2.10 Untargeted metabolomics assessment of the fecal sample

According to previous methods (Li et al., 2022), methanol/chloroform (3:1) and the internal standard solution were added to 10 mg of the stool sample, homogenized for 5 min, and centrifuged at 13,200 g at 4 °C for 15 min. A fraction of the supernatant was then diluted in LC-MS-grade water to yield a solution containing 53% methanol. Subsequently, the samples were transferred to fresh Eppendorf tubes and centrifuged again following the same procedure. Finally, the supernatants were injected for the LC-MS/MS system analysis.

UHPLC-MS/MS analyses were performed using a Vanquish UHPLC system (Thermo Fisher, Germany) coupled with an Orbitrap Q Exactive TM HF-X mass spectrometer (Thermo Fisher, Germany). The samples were loaded onto a Hypersil Gold column (100 × 2.1 mm, 1.9 μm) using a linear gradient of 12 min at a flow of 0.2 mL/min. In the positive polarity mode, the mobile phases used were eluent A (0.1% FA in water) and eluent B (methanol). The eluents for the negative polarity mode were eluent A (5 mM ammonium acetate, pH 9.0) and eluent B (methanol). The solvent gradient was set as follows: 2% B, 1.5 min; 2%–85% B, 3 min; 85%–100% B, 10 min; 100%–2% B, 10.1 min; and 2% B, 12 min. The Q Exactive TM HF-X mass spectrometer was operated in both positive and negative polarity modes. The settings included a spray voltage of 3.5 kV, a capillary temperature of 320 °C, a sheath gas flow of 35 psi, an auxiliary gas flow rate of 10 L/min, an S-lens RF level set to 60, and an auxiliary gas heater temperature of 350 °C. Finally, data analysis was performed using ChromaTOF software. Compound identification was performed using the in-house library containing over 1,000 mammalian metabolite standards and online available libraries (National Institute of Standards and Technology).

### 2.11 Statistical analysis

All statistical analyses were conducted using GraphPad Prism 10.0 software (Prism Inc., San Diego, CA, United States). All experimental data were expressed as the mean ± SD. Statistical analysis was carried out using SPSS 20.0 software in a one-way analysis of variance (ANOVA), followed by the least significant difference (LSD) *post hoc* test or Dunnett’s T3 test for comparison of multiple groups. Differences were considered statistically significant at *p* values of ^*^ *p <* 0.05, ^**^ *p <* 0.01, and ^***^ *p <* 0.001.

## 3 Results

### 3.1 Ginseng and FMT attenuate age-associated oxidative stress damage

Previous studies have demonstrated that ginseng possesses antioxidant, anti-inflammatory, and neuroprotective properties, which contribute to its anti-aging effects (Xu et al., 2023). However, the precise effects and underlying mechanisms of ginseng in anti-aging are not yet fully understood. The effects of ginseng on the fecal microbiota of the mice treated with ginseng and age-associated oxidative stress injury were hereby investigated, and the anti-aging effect of the ginseng extract by intragastric administration or transplantation of ginseng mouse fecal bacteria was observed. Upon 1-week acclimation of all mice, 20-month-old aged mice were categorized into four groups randomly: the aged group (aged), the aged + abx group (Abx), the aged + ginseng group (ginseng), and the aged + ginseng + FMT group (ginseng + FMT). Subsequently, the ginseng group mice were administered ginseng extract via gavage for 9 weeks, while the aged control group mice received the vehicle only. The Abx group and the ginseng + FMT group mice underwent antibiotic treatment. Following this, the ginseng + FMT group mice were given fecal material transplanted from the ginseng group mice (repeated four times for 4 weeks, once a week). All the mice were housed and euthanized at the end of the eighth week (Figure 1A). The body weights of all the mice remained stable throughout the experiment (Figure 1B). Detailed body weight information is provided in Supplementary Material Data S2. In addition, previous research indicated that in aged mice, shorter telomere lengths are associated with a reduced capacity for cellular replication (Gampawar et al., 2022). In the present study, the ginseng group exhibited a significant increase in the telomere length in brain tissues compared to the aged group (*p* < 0.001), suggesting that ginseng helps enhance the replication capacity of cells in aged mice (Figure 1C). Moreover, previous research suggested that oxidative stress was closely associated with brain aging (Yang et al., 2014). Herein, the key oxidative stress indexes associated with aging, antioxidant defense enzymes (SOD, CAT, and GSH-PX), and total antioxidant capacity (T-AOC) in the hippocampus were analyzed, and the results showed increased concentrations in the ginseng group and ginseng + FMT group mice compared to the aged group mice (*p <* 0.001 and *p <* 0.01, respectively) (Figure 1D). However, the levels of ROS, MDA, AGEs, and MAO-B showed descending concentrations in the ginseng group and ginseng + FMT group mice compared to the aged group mice (*p <* 0.001, *p <* 0.05, and *p <* 0.01, respectively) (Figure 1D). These data demonstrated that ginseng and FMT treatment significantly reversed these disorders of oxidative stress indexes associated with aging.

### 3.2 Ginseng and FMT alleviate age-associated systemic inflammation and immune disorder

**FIGURE 2:**
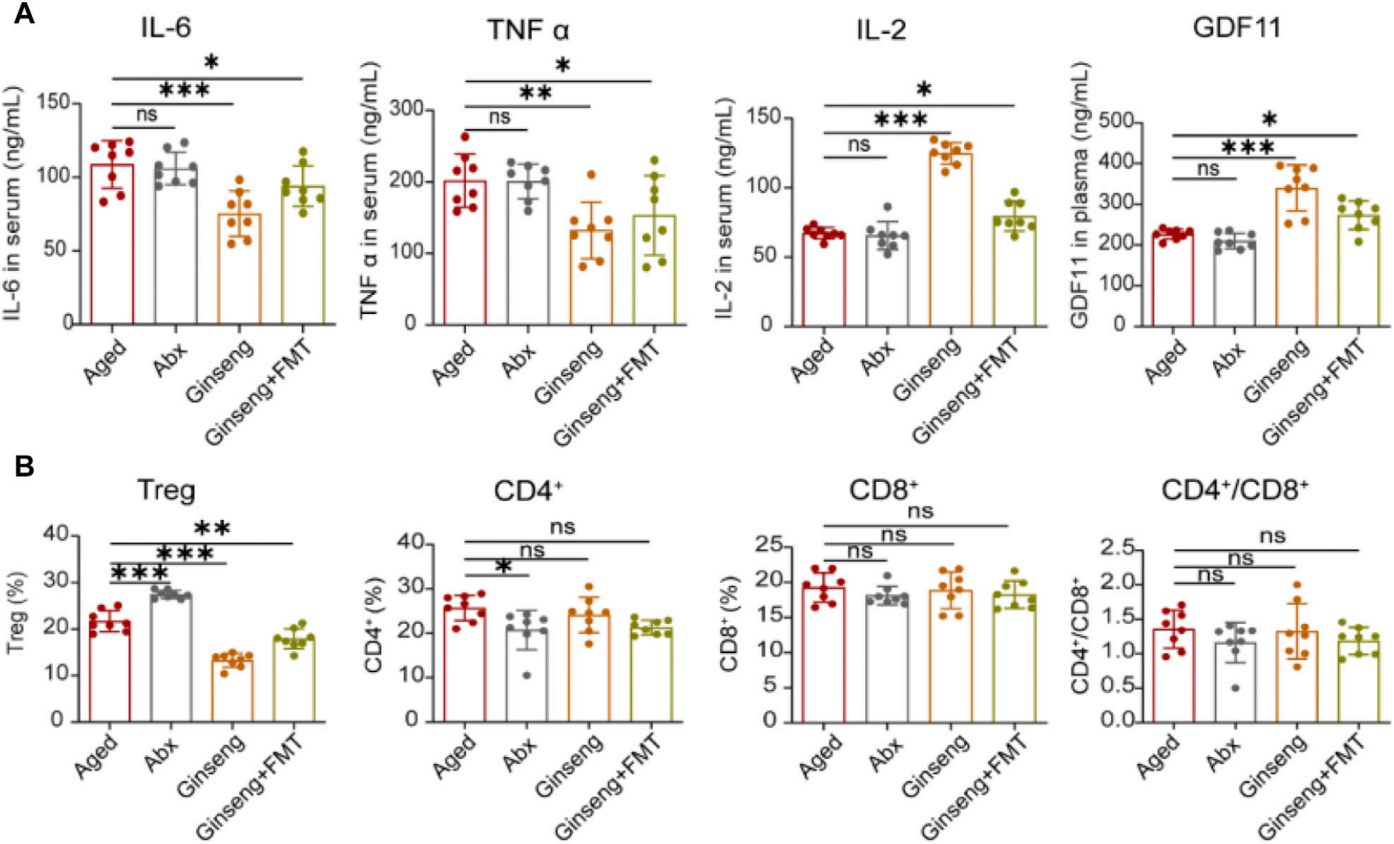
Treatment with ginseng and FMT reduced age-associated inflammation and reversed immune disorder in mice undergoing natural aging. **(A)** Evaluation of inflammation cytokines of IL-6, TNF-α, and IL-2 in serum and GDF11 in plasma with the ELISA method. Data are shown as the mean ± SD, n = 8 per group. Significance was determined by one-way ANOVA, followed by the LSD *post hoc* test or Dunnett’s T3 test for comparison of multiple groups; ^*^ *p <* 0.05, ^**^ *p <* 0.01, and ^***^ *p <* 0.001. **(B)** Flow analysis of the proportion of Treg cells in the spleen and the CD4^+^ and CD8^+^ cells in the blood. CD4^+^/CD8^+^ cells were also calculated. Data are shown as the mean ± SD, n = 8 per group. Significance was determined by one-way ANOVA, followed by the LSD *post hoc* test or Dunnett’s T3 test for the comparison of multiple groups; ^*^ *p <* 0.05, ^**^ *p <* 0.01, and ^***^ *p <* 0.001.

Inflammation is a damage major factor during natural aging, and the anti-inflammatory effects of ginseng have been verified in previous research (Lee et al., 2014; Li et al., 2023). Consistent with the results of previous research, the present investigation results demonstrated that compared to the aged group mice, the treatment with ginseng and FMT decreased the serum concentrations of IL-6 and TNF-α, while IL-2 was increased, indicating that ginseng and FMT had effects on reversing inflammation (*p <* 0.001, *p <* 0.05, and *p <* 0.01) (Figure 2A). In addition, previous studies demonstrated the important role of GDF11 in anti-aging by protecting bodies (Egerman and Glass, 2019). In the present study, the aged mice receiving ginseng or ginseng mouse donor microbiota effectively increased the plasma concentrations of GDF11 compared to those receiving the vehicle (*p <* 0.001 and *p <* 0.05) (Figure 2A). Furthermore, an increase in peripheral T cells and Treg cells in aged mice has been linked to inflammatory responses, as per previous research (Alam et al., 2020; Møller et al., 2022). Herein, the cell proportion of Treg was decreased in the ginseng and FMT group mice compared to the aged group mice (*p <* 0.001 and *p <* 0.01, respectively) (Figure 2B). Collectively, these data indicated that the treatment with ginseng and FMT conferred systemic protection by the attenuation of excessive inflammatory and immune responses.

### 3.3 Ginseng and FMT reverse age-associated abnormal protein expression

**FIGURE 3:**
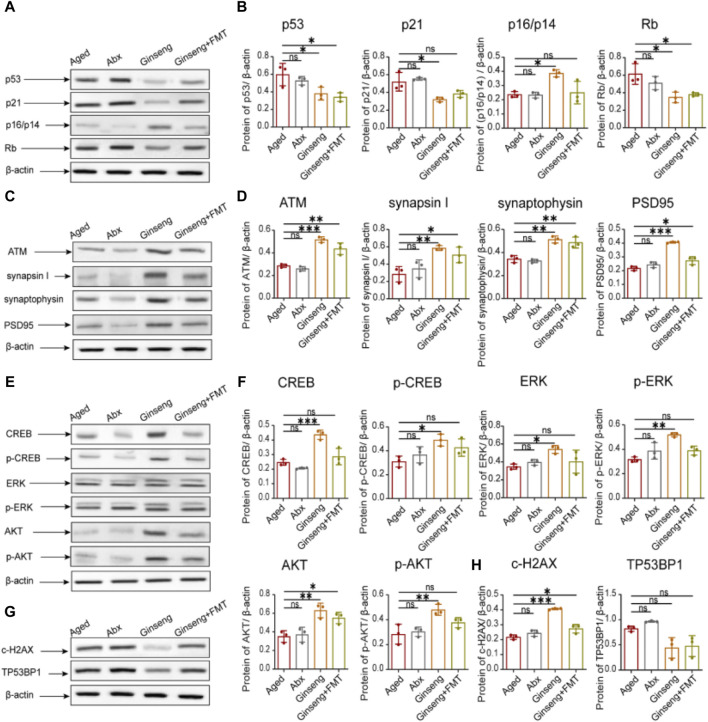
Protective effects of ginseng and FMT in mice against age-associated protein expression levels. **(A,B)** Quantified protein levels of cell cycle arrest-related proteins of p53, p21, p16/p14, and Rb in mouse hippocampus tissues. Data are shown as the mean ± SD, n = 3 per group. Significance was determined by one-way ANOVA, followed by the LSD *post hoc* test or Dunnett’s T3 test for the comparison of multiple groups; ^*^ *p <* 0.05, ^**^ *p <* 0.01, and ^***^ *p <* 0.001. **(C,D)** Quantified protein levels of the DNA damage-related protein ATM and the cognitive-related proteins synapsin I, synaptophysin, and PSD95 in mouse hippocampus tissues. Data are shown as the mean ± SD, n = 3 per group. Significance was determined by one-way ANOVA, followed by the LSD *post hoc* test or Dunnett’s T3 test for the comparison of multiple groups; ^*^ *p <* 0.05, ^**^ *p <* 0.01, and ^***^ *p <* 0.001. **(E,F)** Quantified protein levels of the cell senescence-related proteins CREB, p-CREB, ERK, p-ERK, AKT, and p-AKT in mouse hippocampus tissues. Data are shown as the mean ± SD, n = 3 per group. Significance was determined by one-way ANOVA, followed by the LSD *post hoc* test or Dunnett’s T3 test for the comparison of multiple groups; ^*^ *p <* 0.05, ^**^ *p <* 0.01, and ^***^ *p <* 0.001. **(G,H)** Quantified levels of the c-H2AX and TP53BP1 proteins in bone marrow mesenchymal stem cells. Data are shown as the mean ± SD, n = 3 per group. Significance was determined by one-way ANOVA, followed by the LSD *post hoc* test or Dunnett’s T3 test for the comparison of multiple groups; ^*^ *p <* 0.05, ^**^ *p <* 0.01, and ^***^ *p <* 0.001.

To further explore the molecular changes in protein expression associated with natural aging, the age-associated protein expression was hereby tested. As indicated by the results, compared to the aged group, the cell cycle arrest-related protein expression p53, p21, and Rb was decreased in the hippocampus tissues of the ginseng and ginseng + FMT group mice, while p16/p14 was increased in the ginseng group (*p <* 0.05) (Figures 3A,B). Meanwhile, compared to the aged group, the DNA damage-related protein expression ATM and the cognition-related protein expressions synapsin I, synaptophysin, and PSD95 were all increased in the hippocampus tissues of the ginseng and ginseng + FMT group mice (*p <* 0.001, *p <* 0.01, and *p <* 0.05) (Figures 3C,D). Similarly, compared to the aged group, the cell senescence-related protein expression of CREB, p-CREB, ERK, p-ERK, AKT, and p-AKT was increased in the hippocampus tissues of the ginseng group mice (*p <* 0.001, *p <* 0.05, and *p <* 0.01) (Figures 3E,F). Furthermore, the c-H2AX protein expression in bone marrow mesenchymal stem cells of the ginseng and ginseng + FMT group mice was increased (*p <* 0.001 and *p <* 0.05) (Figures 3G,H). In short, these data suggested that ginseng promoted DNA damage repair, reversed cell cycle arrest, and inhibited stem cell senescence, thus enhancing the mouse cognitive function during natural aging.

### 3.4 Ginseng and FMT improve the aging fecal flora composition while repairing age-associated damage

**FIGURE 4:**
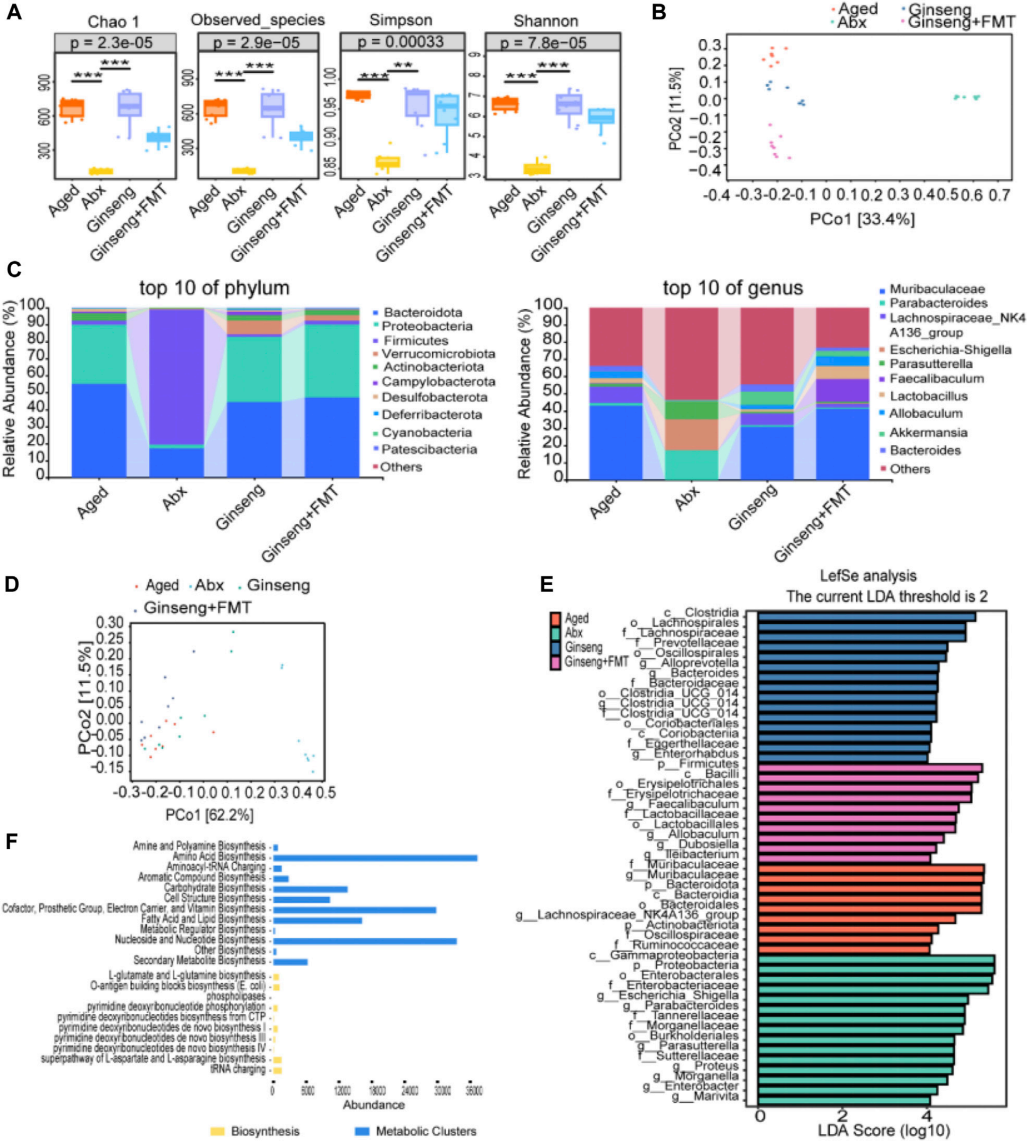
Fecal microbiota structure changes in aged mice following the treatment with ginseng and FMT. **(A)** Comparison of the fora alpha diversity indices. Fora alpha diversity indexes include Chao1, observed species, and Simpson and Shannon indexes, n = 8 per group. **(B)** Principal coordinates analysis (PCoA) of beta diversity. Each dot in the diagram represents a sample, and different colored dots indicate different groupings. The percentages in the brackets represent the percentage of the sample difference data (distance matrix) that can be explained by the corresponding axis. **(C)** Effects of the treatment with ginseng and FMT on the abundance of the fecal microbiota in mice, n = 8 per group. The top 10 changes in the composition of the phylum level (left) and genus level (right) in mouse fecal microbiota are shown. **(D)** OPLS-DA of the species difference between groups. Each dot represents a sample, and different colored dots indicate different groups. The closer the projection distance between two points on the coordinate axis, the more similar the species abundance composition between the two samples in the corresponding dimension. **(E)** High-dimensional biomarkers of histogram of LDA. LDA value distribution histogram revealed using LEfSe software. Species with LDA scores >4 are statistically different; the length of the LDA score represents the impact size of the different species. The deep-red histogram indicates the aged group, the green histogram indicates the Abx group, the blue histogram indicates the ginseng group, and the peach histogram indicates the ginseng + FMT group. n = 8 per group. **(F)** Potential metabolic pathway statistics. The yellow histogram indicates the biosynthesis pathways, and the blue histogram indicates the metabolic cluster pathways.

To further identify specific changes in the fecal microbiota among different groups, 16S rDNA sequencing of all mice was performed. The results indicated that the box diagram of Chao1, observed species, and Shannon and Simpson indexes intuitively showed higher diversity of fecal flora of mice in the ginseng group than in the aged and Abx groups (*p* < 0.001 and *p* < 0.01) (Figure 4A). In addition, principal coordinates analysis (PCoA) showed that the composition and structure of fecal flora in the four groups were significantly different. In particular, in the Abx group, distinct clusters of microbial species were formed on the right of the main horizontal axis, while the ginseng + FMT group presented clusters on the lower left (Figure 4B). The results of relative species abundance of the phylum-level analysis showed that the top 10 relatively high proportions varying greatly among the groups were *Bacteroidota, Proteobacteria, Firmicutes, Verrucomicrobiota, Actinobacteriota, Campylobacterota, Desulfobacterota, Deferribacterota, Cyanobacteria,* and *Patescibacteria* (Figure 4C)*.* In addition, the genus-level analysis showed that the top 10 relatively high proportions varying greatly among groups were Muribaculaceae*, Parabacteroides,* Lachnospiraceae*_NK4A136_group, Escherichia–Shigella, Parasutterella, Faecalibaculum, Lactobacillus, Allobaculum, Akkermansia,* and *Bacteroides* (Figure 4C). Furthermore, OPLS-DA revealed significant differences in the microbial species composition between the groups (Figure 4D). Next, LEfSe was used to interpret high-dimensional biomarkers of fecal samples, and high-dimensional biomarkers were shown as the histogram of LDA. Genus species with LDA scores >4 were statistically different in high-dimensional biomarkers, and the top 10 were Muribaculaceae*, Escherichia–Shigella, Parabacteroides, Faecalibaculum,* Lachnospiraceae*_NK4A136_group, Parasutterella, Proteus, Morganella, Allobaculum,* and *Alloprevotella* (Figure 4E). Finally, the potential metabolic pathways enriched by these different bacterial genera were predominantly associated with amino acid biosynthesis, nucleoside and nucleotide biosynthesis, cofactor, prosthetic group, electron carrier, vitamin biosynthesis, etc. (Figure 4F).

### 3.5 Untargeted metabolomics assessment results of the anti-aging effects of ginseng and FMT

**FIGURE 5:**
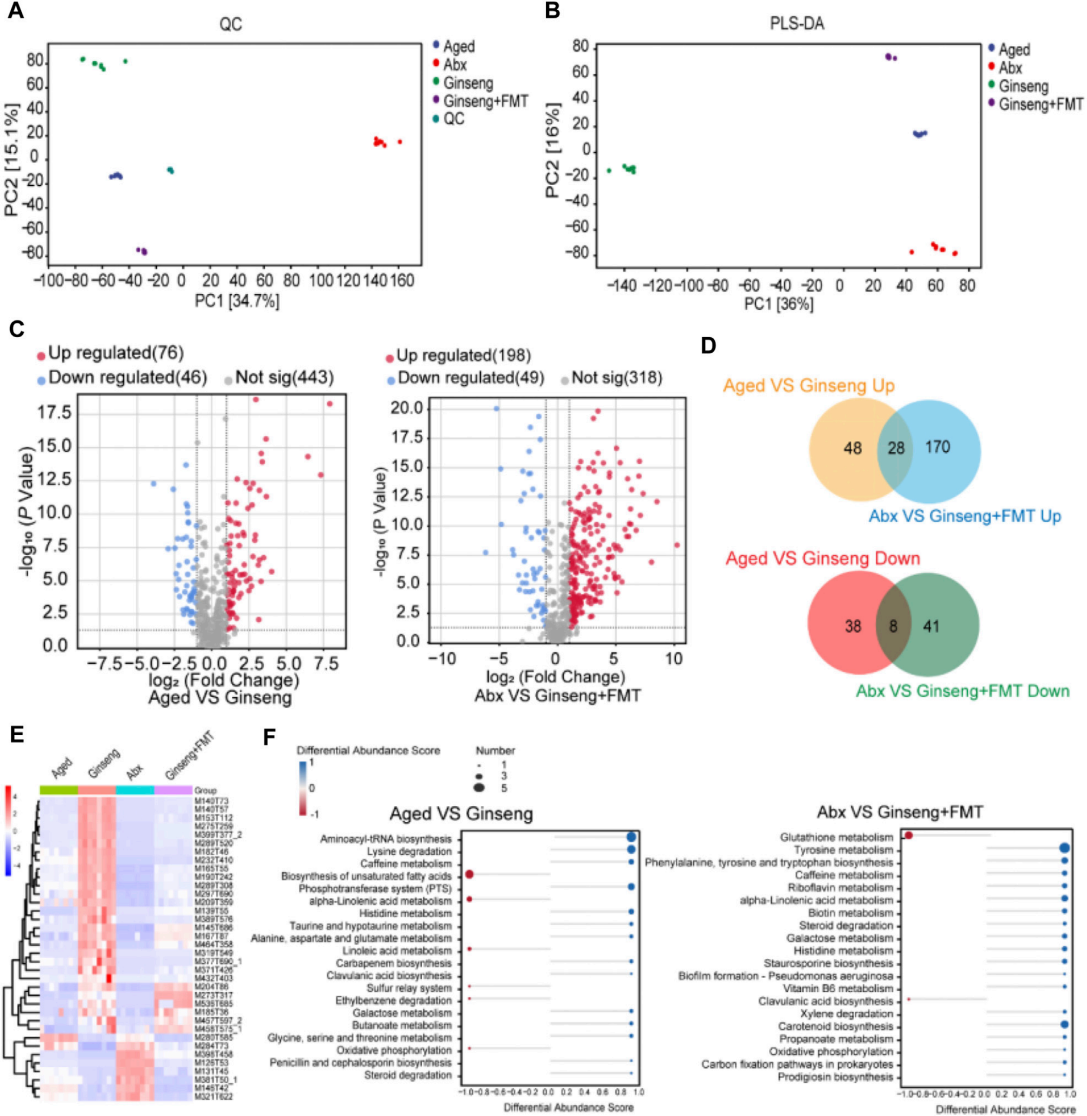
Untargeted metabolomics assessment of fecal samples of aged mice following the treatment with ginseng and FMT. **(A)** Overall PCA diagram of QC. PC1 represents principal component 1, and PC2 represents principal component 2; each dot represents a sample, and different colored dots indicate different groups. The peaks extracted from all experimental samples and QC samples were analyzed by PCA. n = 8 per group. **(B)** Multivariate statistical PLS-DA. n = 8 per group. **(C)** Volcano plot of differentially expressed metabolites. n = 8 per group. **(D)** Upper: Venn diagram showing the overlap between significantly upregulated metabolites in aged vs ginseng group mice and significantly upregulated metabolites in Abx vs ginseng + FMT group mice. Lower: Venn diagram showing the overlap between significantly downregulated metabolites in aged vs ginseng group mice and significantly downregulated metabolites in Abx vs ginseng + FMT group mice. Differentially expressed metabolites were identified with a cutoff of |fold change|>2 and adjusted *p*-value<0.05. **(E)** Heatmap of the expression levels of metabolites differentially expressed in the aged or Abx group and rescued in the ginseng or ginseng + FMT group. The expression level for each metabolite is represented by a color range from blue (low) to red (high). **(F)** Enrichment result of difference abundance analysis. Left, differential material enrichment analysis of aged vs ginseng mice. Right, differential material enrichment analysis of Abx vs ginseng + FMT mice. The Y-axis represents the name of the metabolic pathway, and the X-axis coordinates represent the DA score. The DA score is the overall total change in all metabolites in the metabolic pathway. A score of 1 indicates upregulated expression of all identified metabolites in this pathway, and −1 indicates downregulated expression of all identified metabolites in this pathway. The length of the line segment represents the absolute value of the DA score, and the size of the dot at the end of the line segment represents the number of metabolites in the pathway. The larger the dot, the greater the number of metabolites. The depth of the color of the line segment and dot is proportional to the DA score value. The darker the red, the more inclined the overall expression of the channel is to upregulate, and the darker the blue, the more inclined the overall expression of the channel is to downregulate.

The results of the untargeted metabolomics assessment of fecal samples in this study are shown in Figure 5. The clustering of QC samples on the PCA chart demonstrated the consistency and reliability of the data in this study (Figure 5A). In addition, the multivariate statistical PLS-DA indicated significant metabolic differences among the groups (Figure 5B). The volcano plot results revealed that ginseng treatment led to the upregulation of 76 metabolites and the downregulation of 46 metabolites compared to the aged group (Figure 5C, left). Furthermore, the volcano plot results indicated that after receiving the ginseng + FMT treatment, there was a significant upregulation of 198 differentially expressed metabolites and a downregulation of 49 metabolites compared to the Abx group (Figure 5C, right). In addition, 28 differentially expressed metabolites were upregulated upon the treatment with FMT, while 8 differentially expressed metabolites were downregulated upon the treatment with FMT (Figure 5D). Moreover, the heatmap of the expression levels of 36 kinds of metabolites differentially demonstrated that imidazolepropionic acid, 3-hydroxypicolinic acid, xanthine, N2-succinyl-L-arginine, and tigogenin were significantly increased upon the treatment with ginseng, while N-acetyl-D-galactosamine, garbanzol, beta-carotene, 3,4-dihydroxymandelic acid, and ursolic acid were significantly increased upon the treatment with ginseng or ginseng + FMT (Figure 5E). Finally, after treating with ginseng, the differential material enrichment analysis results showed that in the biosynthesis of unsaturated fatty acids and alpha-linolenic acid metabolism and linoleic acid metabolism pathways, the identified metabolites were downregulated, while in aminoacyl-tRNA biosynthesis, lysine degradation, and phosphotransferase system (PTS) pathways, the identified metabolites were upregulated (Figure 5F, left). Upon the treatment with FMT, in glutathione metabolism and clavulanic acid biosynthesis pathways, the identified metabolites were downregulated, while in tyrosine metabolism and phenylalanine, tyrosine, tryptophan, and carotenoid biosynthesis pathways, the identified metabolites were upregulated (Figure 5F, right).

### 3.6 Spearman’s correlation analysis results of the fecal bacteria and metabolites

**FIGURE 6:**
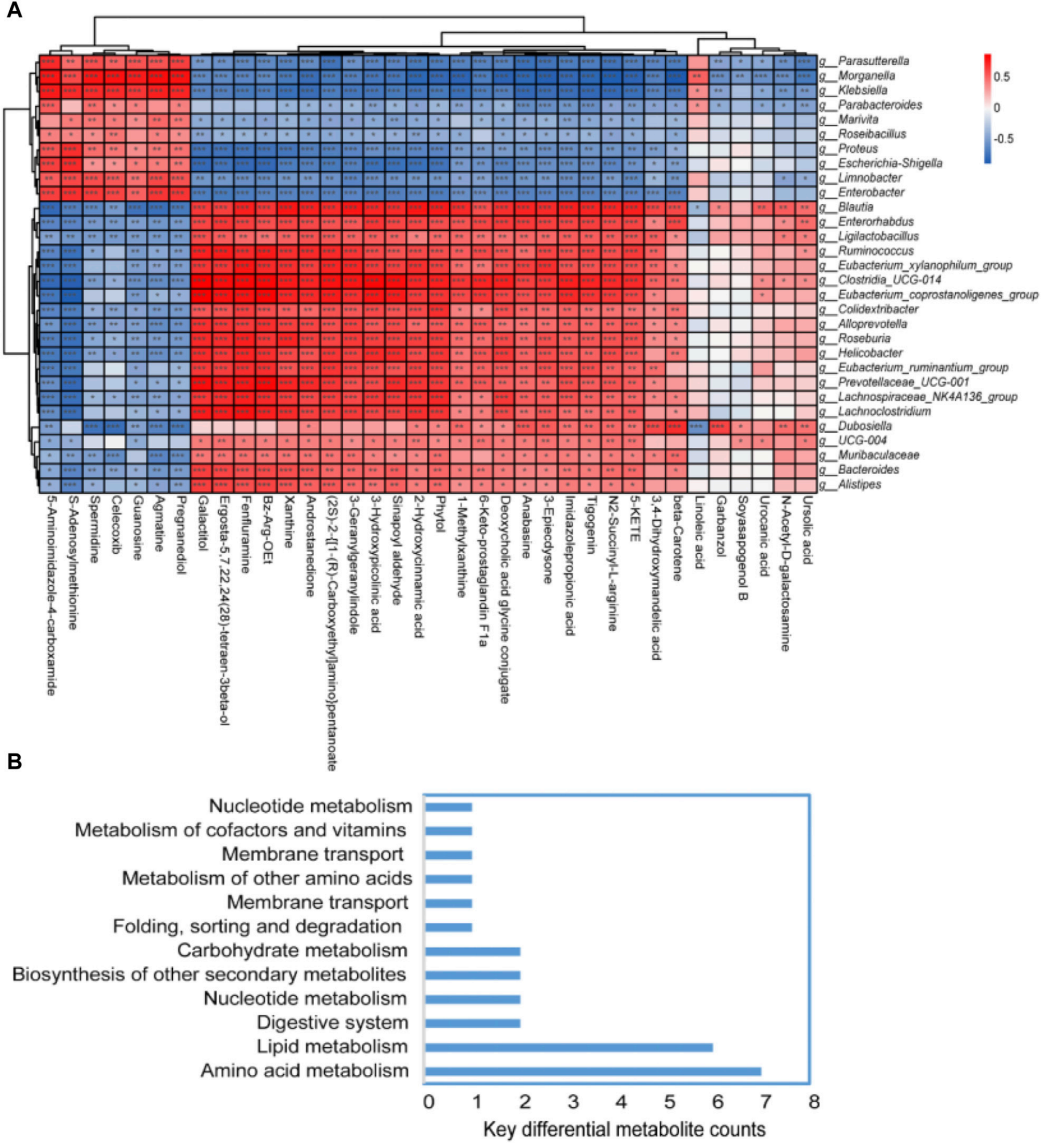
Spearman’s correlation analysis of the fecal flora and fecal metabolites. **(A)** Heatmap of the correlation between bacterial species and metabolomics test results. If the correlation between the two is positive, it is shown in red; otherwise, it is shown in blue. The depth of the color indicates the strength of the correlation. “*” indicates bacterial genus metabolites with significant associations (*p* < 0.05). **(B)** Key metabolism pathways of key differential metabolites.

The top 50 differential bacterial genera at the genus level and 36 key differential metabolites were identified and screened for correlation analysis. Figure 6A shows the results, which revealed a significant positive correlation between the bacteria of *Parasutterella, Morganella, Klebsiella, Parabacteroides, Marivita, Roseibacillus, Proteus, Escherichia–Shigella, Limnobacter,* and *Enterobacter* and the metabolite of 5-aminoimidazole-4-carboxamide, S-adenosylmethionine, spermidine, celecoxib, guanosine, agmatine, and pregnanediol. Similarly, a significant positive correlation was observed between the bacteria of *Blautia, Enterorhabdus, Ligilactobacillus, Ruminococcus, Eubacterium_xylanophilum_group, Clostridia_UCG-014, Eubacterium_coprostanoligenes_group, Colidextribacter, Alloprevotella, Roseburia, Helicobacter,* and *Eubacterium_ruminantium_group* and the metabolite of galactitol, ergosta-5,7,22,24(28)-tetraen-3beta-ol, fenfluramine, Bz-Arg-OEt, xanthine, androstanedione, (2S)-2-{[1-(R)-carboxyethyl]amino}pentanoate, 3-geranylgeranyl indole, 3-hydroxypicolinic acid, sinapoyl aldehyde, 2-hydroxycinnamic acid, phytol, and 1-methylxanthine. Furthermore, these significantly different bacteria and metabolites were mainly enriched in the pathways of amino acid metabolism, lipid metabolism, nucleotide metabolism, etc. (Figure 6B). Detailed information about the 36 key differential metabolites is given in Table 1.

**TABLE 1:** Information of 36 key differential metabolites.

ID	Name	mz	rt	exact_mass	ppm	Formula	precursor_type
M126T53	5-Aminoimidazole-4-carboxamide	126.05	52.9	126.05	0.638277	C_4_H_6_N_4_O	[M]^+^
M131T45	Agmatine	131.13	45	130.12	0.332338	C_5_H_14_N_4_	[M + H]^+^
M146T42	Spermidine	146.17	42.3	145.16	0.00424	C_7_H_19_N_3_	[M + H]^+^
M280T585	Linoleic acid	280.26	584.9	280.24	1.328038	C_18_H_32_O_2_	[M]^+^
M284T73	Guanosine	284.10	73.4	283.09	2.73145	C_10_H_13_N_5_O_5_	[M + H]^+^
M321T622	Pregnanediol	321.31	621.7	320.27	2.729043	C_21_H_36_O_2_	[M + H]^+^
M381T50_1	Celecoxib	381.08	49.6	381.08	3.148969	C_17_H_14_F_3_N_3_O_2_S	[M]^+^
M398T458	S-Adenosylmethionine	398.24	458.5	398.14	1.308606	C_15_H_22_N_6_O_5_S	[M]^+^
M139T55	Urocanic acid	139.05	54.6	138.04	1.265732	C_6_H_6_N_2_O_2_	[M + H]^+^
M140T57	3-Hydroxypicolinic acid	140.03	57.1	139.03	0.064455	C_6_H_5_NO_3_	[M + H]^+^
M140T73	Imidazolepropionic acid	140.05	72.9	140.06	0.150089	C_6_H_8_N_2_O_2_	[M]^+^
M145T686	Anabasine	144.98	686	162.12	7.527574	C_10_H_14_N_2_	[M + H-H2O]^+^
M153T112	Xanthine	153.04	111.6	152.03	5.070573	C_5_H_4_N_4_O_2_	[M + H]^+^
M165T55	2-Hydroxycinnamic acid	165.05	55.2	164.05	1.053453	C_9_H_8_O_3_	[M + H]^+^
M167T87	1-Methylxanthine	167.06	87.2	166.05	0.001881	C_6_H_6_N_4_O_2_	[M + H]^+^
M182T46	Galactitol	182.09	46.4	182.08	2.516189	C_6_H_14_O_6_	[M]^+^
M185T36	3,4-Dihydroxymandelic acid	184.98	36.2	184.04	12.78595	C_8_H_8_O_5_	[M + H]^+^
M190T242	(2S)-2-{[1-(R)-Carboxyethyl]amino}pentanoate	190.11	241.7	189.10	1.451811	C_8_H_15_NO_4_	[M + H]^+^
M204T86	N-Acetyl-D-galactosamine	204.09	86.4	221.09	20.25611	C_8_H_15_NO_6_	[M + H-H2O]^+^
M209T359	Sinapoyl aldehyde	209.08	359.5	208.07	1.241892	C_11_H_12_O_4_	[M + H]^+^
M232T410	Fenfluramine	232.13	409.9	231.12	1.38137	C_12_H_16_F_3_N	[M + H]^+^
M273T317	Garbanzol	273.08	317.2	272.07	0.087888	C_15_H_12_O_5_	[M + H]^+^
M275T259	N2-succinyl-L-arginine	275.14	258.6	274.13	15.35246	C_10_H_18_N_4_O_5_	[M + H]^+^
M289T308	Bz-Arg-OEt	289.17	308.1	306.17	13.25901	C_15_H_22_N_4_O_3_	[M + H-H2O]^+^
M289T520	Androstanedione	289.21	519.9	288.21	6.140735	C_19_H_28_O_2_	[M + H]^+^
M297T690	Phytol	297.31	689.6	296.31	3.282722	C_20_H_40_O	[M + H]^+^
M319T549	5-KETE	319.23	548.5	318.22	4.310434	C_20_H_30_O_3_	[M + H]^+^
M371T426	6-Keto-prostaglandin F1a	371.26	426.2	370.24	1.949464	C_20_H_34_O_6_	[M + H]^+^
M377T690_1	Ergosta-5,7,22,24(28)-tetraen-3beta-ol	377.32	689.6	394.32	1.945314	C_28_H_42_O	[M + H-H2O]^+^
M389T576	3-Geranylgeranyl indole	389.30	576.3	389.31	8.733515	C_28_H_39_N	[M]^+^
M399T377_2	Tigogenin	399.32	376.8	416.33	3.090241	C_27_H_44_O_3_	[M + H-H2O]^+^
M432T403	Deoxycholic acid glycine conjugate	432.31	402.5	449.31	8.868704	C_26_H_43_NO_5_	[M + H-H2O]^+^
M457T597_2	Ursolic acid	457.37	597.1	456.36	5.518507	C_30_H_48_O_3_	[M + H]^+^
M458T575_1	Soyasapogenol B	458.37	574.6	458.38	13.30803	C_30_H_50_O_3_	[M]^+^
M464T358	3-Epiecdysone	464.30	358.4	464.31	3.377588	C_27_H_44_O_6_	[M]^+^
M536T685	Beta-carotene	536.16	684.7	536.44	5.256554	C_40_H_56_	[M]^+^

## 4 Discussion

Aging is closely associated with many common age-related diseases, including Alzheimer’s disease, coronary heart disease, diabetes, tumors, and osteoporosis, all of which seriously affect the quality of life of the elderly (Li X. et al., 2023; Xiao et al., 2023). Age-related cognitive impairment of the brain and gastrointestinal dysfunction caused by intestinal flora disorder are two major problems seriously plaguing elderly patients, making it imperative to address the urgent matter of increasing the healthspan in the elderly. A growing body of evidence has suggested that rejuvenating the aging gut and brain is a key strategy to promote the health of the elderly, and plant extracts have received widespread attention due to their few side effects and significant health benefits for the elderly (Agatonovic-Kustrin et al., 2019; Salem et al., 2020). The recent surge in reports on anti-aging plant extracts highlights their longstanding use and relatively mild side effects, offering promising avenues for mitigating age-related damage. Ginseng, a traditional Chinese medicine with a history spanning over 4,000 years, has been the subject of modern research that suggests its significant anti-aging properties. In accordance with current laws and regulations, ginseng is recognized for its versatility, being applicable not only in pharmaceuticals but also in health foods, culinary uses, and skincare products. This underscores the expansive potential and promising future of ginseng in the realm of anti-aging (de Oliveira et al., 2022; Su et al., 2023). However, the precise role of ginseng in the homeostasis of the protection of the aging brain and gut has not yet been identified. Furthermore, there is a notable gap in research concerning the impact and underlying mechanisms of ginseng on the delay of age-related cognitive decline in naturally aged mice, specifically through the lens of the microbiota–gut–brain axis. In the present study, a long-term dietary intake of ginseng for 9 weeks was designed to determine the effects of ginseng on age-related brain and gut aging. Furthermore, it was hypothesized that ginseng-mediated changes in the gut microbiota could potentially enhance intestinal balance and mitigate brain deterioration in mice experiencing natural aging. To explore this possibility, 20-month-old naturally aged mice were orally administered ginseng and served as fecal flora donors. FMT was subsequently performed to explore the mechanisms of ginseng in anti-aging from the aspect of the fecal microbiota and metabolites.

The pathological mechanisms of aging are complex and diverse, among which the free radical theory and mitochondrial theory imply that the damage caused by oxygen free radicals is one of the main reasons for cell aging (Zhang et al., 2023a). Oxidative stress is a negative effect of free radicals in the body, which will gradually increase throughout the life cycle of natural aging, and the accumulated oxidative stress will contribute to cognitive decline and, ultimately, neurodegenerative diseases (Hajam et al., 2022). Previous research indicates that the oxidative stress of brain aging is mainly manifested by the increase in oxidative stress markers ROS, MDA, AGEs, and MAO-B levels, as well as the decrease in antioxidant markers SOD, CAT, GSH-PX, and T-AOC levels in the hippocampus (Chaudhary et al., 2023). Meta-analytic studies underscore the pivotal role of the telomere length in enhancing the brain structure and cognitive function (Gampawar et al., 2022). Aging leads to immune system dysregulations, including Treg cell imbalances in the spleen and fluctuations in CD4^+^ and CD8^+^ T cells in the blood. Furthermore, there is an inflammatory response disorder marked by altered levels of IL-6, TNF-α, IL-2, and GDF11 in the serum (Tao et al., 2023). In line with previous research, this study discovered that the anti-aging effects of ginseng included mitigating brain damage through its antioxidant, anti-inflammatory, and immune-modulating properties. Specifically, ginseng decreased the levels of oxidative stress markers of ROS, MDA, AGEs, and MAO-B and pro-inflammatory factors of IL-6 and TNF-α while increasing the levels of antioxidant markers of SOD, CAT, GSH-PX, and T-AOC, anti-inflammatory factors of IL-2 and GDF11, and immune levels of Treg cell proportions of aging mice. In addition, ginseng treatment resulted in the decreased expression of cell cycle arrest-related proteins p53, p21, and Rb in hippocampal tissues, whereas the expression of p16/p14 was found to be increased. Meanwhile, the DNA damage-related protein expression ATM and the cognitive-related protein expression synapsin I, synaptophysin, and PSD95 were increased in the hippocampus tissues of ginseng-treated mice. Similarly, the cell senescence-related protein expression of CREB, p-CREB, ERK, p-ERK, AKT, and p-AKT was increased in the hippocampus tissues of ginseng-treated mice. Furthermore, the c-H2AX protein expression in the bone marrow mesenchymal stem cells of ginseng-treated mice was increased. Collectively, these results suggested that dietary ginseng alleviated brain damage in naturally aging mice via anti-oxidative stress, inflammation, and immune regulation, enhanced the mouse cognitive function by promoting DNA damage repair, reversed cell cycle arrest, and inhibited the stem cell senescence.

Accumulating evidence has suggested that ginsenosides can be used as a potential novel therapeutic agent or nutraceutical additive in anti-inflammation and anti-aging (Cheng et al., 2005; Chen et al., 2014). Ginsenoside Re has been demonstrated to have anti-aging effects by attenuating memory impairments, involving the regulation of the angiotensin II AT1 receptor, Nrf2, GPx-1, PAFR, NFkappaB, etc. (Nguyen et al., 2022; Shin et al., 2023). Moreover, research has confirmed the anti-inflammatory and immunomodulatory effects of ginsenoside Rg1 in ameliorating experimental colitis by regulating the balance of intestinal flora (Long et al., 2022). Emerging evidence has suggested that the gut microbiota plays a critical role in repairing age-related damage during natural aging (Badal et al., 2020; Honarpisheh et al., 2022). Based on the aforementioned results, 20-month-old naturally aged mice orally administered ginseng were hereby used as fecal flora donors, and FMT was performed combined with 16S rDNA sequencing analysis and untargeted metabolomics subsequently to explore the mechanisms of ginseng in anti-aging from the aspect of the microbiota–gut–brain axis. Interestingly, the fecal microbiota from the ginseng group mouse donors demonstrated anti-aging effects similar to ginseng itself in the naturally aging mice. Antibiotic treatment significantly reduced bacterial diversity in the naturally aging mice, suggesting that antibiotics cleared the gut microbiota of the mice to some extent. Moreover, compared to the aged mice, ginseng + FMT induced an increase in the microbiota diversity and the abundance of beneficial microorganisms, including Muribaculaceae*, Faecalibaculum,* and Lachnospiraceae*_NK4A136_group*, while decreasing the abundance of potential pathogenic bacteria, such as *Bacteroidota, Proteobacteria,* and *Firmicutes*. Furthermore, research has indicated that Lachnospiraceae could be a critical signature of longevity and a beneficial microbiome in anti-aging (DeJong et al., 2020). Furthermore, evidence has suggested that the relative abundance of Lachnospiraceae*_NK4A136* is positively correlated with the production of anti-inflammatory cytokines and the levels of SCFAs but negatively correlated with pro-inflammatory cytokines (Hou et al., 2023). Otherwise, after treating with ginseng or FMT, the metabolomics results showed that metabolites of 3,4-dihydroxymandelic acid, N-acetyl-D-galactosamine, garbanzol, ursolic acid, soyasapogenol B, and β-carotene were upregulated compared to the aged mice. Previous research has indicated that ursolic acid exerts anti-aging effects by reducing oxidative stress, protecting various organs, and promoting the rejuvenation of skeletal muscle (Bakhtiari et al., 2015; Bahrami and Bakhtiari, 2016; Gharibi et al., 2018; Kazemi Pordanjani et al., 2023). Reports have also suggested that β-carotene exerts an anti-aging effect by regulating the KAT7-P15 signaling axis, inflammation, and oxidative stress (Zheng et al., 2022). These lines of evidence indicated that changes in the gut microbiota and its metabolites, resulting from ginseng intervention, played a pivotal role in the anti-aging process.

A detailed Spearman’s correlation analysis was conducted to investigate the interplay between the fecal microbiota and metabolites, illuminating the role of the microbiota–gut–brain axis in the context of ginseng treatment and FMT in naturally aging mice. The data indicated a significant positive correlation between the bacteria of *Parasutterella, Morganella, Klebsiella, Parabacteroides, Marivita, Roseibacillus, Proteus, Escherichia–Shigella, Limnobacter,* and *Enterobacter,* and the metabolite of 5-aminoimidazole-4-carboxamide, S-adenosylmethionine, spermidine, celecoxib, guanosine, agmatine, and pregnanediol. Similarly, a significant positive correlation was observed between the bacteria of *Helicobacter,* Lachnospiraceae*_NK4A136_group, Lachnoclostridium, Dubosiella,* and *Bacteroides* and the metabolite of xanthine, β-carotene, linoleic acid, ursolic acid, etc. Previous research has indicated that *Enterobacter*-driven novel biosynthesis of tin oxide nanoparticles exerts an anti-aging effect (Rizwan et al., 2023). *Dubosiella* exerts anti-aging effects by mitigating oxidative stress, bolstering vascular endothelial function, and balancing the gut microbiota (Liu et al., 2023). *Bacteroides* can prevent aging-related damage in the heart by regulating T cells and decreasing inflammation (Zhang et al., 2022; Duan et al., 2023). However, *Helicobacter* increases the risk of atherosclerosis in the elderly (Shan et al., 2017; Yamashita et al., 2019). Excessive predominance of pathological species *Lachnoclostridium* in the gut microbiota can increase the production of inflammatory mediators accelerating the aging process (Manchia et al., 2023). In addition, research has suggested that S-adenosylmethionine ameliorates cognitive impairment in brain aging by inhibiting oxidative stress and neuroinflammation (Zhang et al., 2023b). Spermidine impedes brain aging by inducing autophagy, preventing apoptosis, and reducing the accumulation of inflammation (Xu et al., 2020; Yang et al., 2020; Wortha et al., 2023). Guanosine, released by astrocytes, plays a crucial role in modulating astroglial functions and offers neuroprotection during the aging process (Souza et al., 2016). Celecoxib can target pro-inflammatory mediators in the chemoprevention of prostatic disorders during aging (Kido et al., 2017). Linoleic acid can prevent bone loss during aging (Sasaki et al., 2019; Abdulrahman et al., 2023). Conversely, oxidoreductase products of xanthine can accelerate the aging process (Battelli et al., 2020). Furthermore, these significantly different bacteria and metabolites are mainly enriched in the pathways of amino acid metabolism, lipid metabolism, nucleotide metabolism, etc. Amino acid metabolism plays an important role in anti-aging (Roberts et al., 2020; Chen et al., 2022; Henderson et al., 2023). Lipid metabolism plays a crucial role in protecting the heart, liver, and blood vessels during the natural aging process (Johnson and Stolzing, 2019; Mutlu et al., 2021; Li et al., 2022). The increased histamine levels in aged brown fat may be a potential biomarker closely linked to nucleotide metabolism (Mancini et al., 2021). This study suggested that ginseng could potentially mitigate intestinal aging by promoting beneficial bacteria and metabolites and might also slow cognitive decline associated with aging through the circulation of these beneficial elements in the blood. However, future research should involve *in vivo* experiments administering these beneficial microorganisms or metabolites directly to aging mice, as well as clinical trials with ginseng supplementation among elderly individuals, to further investigate the efficacy, mechanisms, and conclusive evidence of the anti-aging properties of ginseng.

## 5 Conclusion

In summary, the present study utilized naturally aging mice treated orally with ginseng as fecal flora donors, followed by FMT. The findings suggested that ginseng might protect the brain by enriching beneficial bacteria of Lachnospiraceae*, Enterobacter*, *Dubosiella*, *Bacteroides,* etc., and metabolites of ursolic acid, β-carotene, S-adenosylmethionine, spermidine, guanosine, celecoxib, linoleic acid, etc., by regulating the microbiota–gut–brain axis. The existence of a link between metabolic biomarkers and the gut microbiota had an important influence on the host. Key microbiota and metabolites were predominantly enriched in pathways such as amino acid metabolism, lipid metabolism, and nucleotide metabolism. This indicated that ginseng might contribute to anti-aging effects through the modulation of these metabolic pathways. This study identified key functional bacteria and potential biomarkers of aging in mice and established a correlation between them. Altogether, these results have important implications for the development of ginseng as an anti-aging protective agent in anti-aging from the aspect of the microbiota–gut–brain axis. However, further preclinical and translational studies should still be conducted to investigate the effects and safety of ginseng before its application in clinical anti-aging treatments.

## Data Availability

The data presented in the study are deposited in the GitHub repository, available at https://github.com/Zystry1/metabolomics-raw-data.git and https://github.com/Zystry1/fecal-microbiota-16S-rRNA-sequencing.git.
