# Supplementary material for: Research on the anti-aging mechanisms of Panax ginseng extract in mice: a gut microbiome and metabolomics approach
Source: Front Pharmacol. 2024 Jun 20;15:1415844. doi: 10.3389/fphar.2024.1415844 (PMC11222675; doi:10.3389/fphar.2024.1415844)
Supplement: Supplementary file 1 [file DataSheet1.docx]

**Supplementary Material Data**

The herbal of ginseng was obtained from Anguo Changda Chinese herbal medicine pieces Co., LTD (Baoding, China) and identified by Professor Hui Li (Institute of Chinese Materia Medica, China Academy of Chinese Medical Sciences, Beijing, China) as the dried roots and rhizomes of *Panax ginseng* C. A. Mey., plants of the acanthopanax family (hereinafter referred to as ginseng). The used ginseng extract in this experiment was prepared according to the following preparation method. We used 200 g of ginseng root adding 1600 mL water, which was boiled for 1.5 hours, and the water extract was drained out. Next, 1600 mL water was added and left to boil for 1.5 hours, after which the remaining water extract was drained out again. All water extract was put together and concentrated to the constant with Rotary Evaporators (TOKYO RIKAKIKAI CO., LTD. Type: N-12108V-WB). Then, the resulting concentrated liquid was frozen at -30 ℃ for 20 minutes, freeze-dried into solid using a freeze-dried machine (Foring Science Instrument Technology Development Co., Ltd, Beijing, China), and crushed into powder for use. Finally, 68 g ginseng extract powder was obtained (the mass ratio between raw ginseng and ginseng extract powder was 200 : 68).

**1. Fingerprinting Methods**

**1.1 Preparation of test solution**

1.1.1 Pharmacopoeia method Take 0.4 g of this ginseng extract powder, weigh accurately, add 50 mL of saturated n-butanol with water, seal tightly, leave overnight, sonicate (power 250 W, frequency 50 kHz) for 30 minutes, filter, discard the initial filtrate, accurately measure 25 mL of the subsequent filtrate, evaporate to dryness in an evaporating dish, dissolve the residue in methanol and transfer to a 5 mL volumetric flask, dilute with methanol to the mark, shake well, filter, and take the subsequent filtrate to obtain.

1.1.2 Methanol method Take 1.0 g of this ginseng extract powder, accurately weigh it, place it in a 25 mL volumetric flask, add about 20 mL of methanol, seal it tightly, and sonicate (power 250 W, frequency 50 kHz) for 30 minutes. Take it out and place it at room temperature, add methanol to the mark, shake well, filter it, and take the filtrate again to obtain it.

1.1.3 Ethanol method Take 1.0 g of this ginseng extract powder, accurately weigh it, place it in a 25 mL volumetric flask, add about 20 mL of 70 % ethanol, close the stopper, and sonicate (power 250 W, frequency 50 kHz) for 30 minutes. Take it out and place it at room temperature, add 70 % ethanol to the mark, shake well, filter, and take the filtrate again to obtain the desired result.

**1.2 Measurement methods**

Using Waters 2695 high-performance liquid chromatography with octadecylsilane bonded silica gel as the filler; Using acetonitrile as the mobile phase A and water as the mobile phase B, perform gradient elution as specified in the table below; The detection wavelength is 203 nm. Mobile phase elution gradient information was shown in **Table**

**S1**.

**Table S1** Mobile phase elution gradient information.

| Time (min) | Mobile phase A (%) | Mobile phase B (%) |
| --- | --- | --- |
| 0-35 | 19 | 81 |
| 35-55 | 19-29 | 81-71 |
| 55-70 | 29 | 71 |
| 70-100 | 29-40 | 71-60 |
| 100-110 | 40-95 | 60-5 |
| 110-111 | 95-19 | 5 |
| 111-120 | 19 | 81 |

**2. Result**

The HPLC detection results are as followings. As results shown in **Table S2**, Ginsenoside Rg1, Ginsenoside Re, and Ginsenoside Rb1 in the pharmacopoeia method were tested as1.33 %, 0.25 %, and 0.36 %, respectively. Besides, Ginsenoside Rg1, Ginsenoside Re, and Ginsenoside Rb1 in the methanol method were tested as1.19 %, 0.23 %, and 0.33 %, respectively. Furthermore, Ginsenoside Rg1, Ginsenoside Re, and Ginsenoside Rb1 in the 70 % ethanol method were tested as1.24 %, 0.24 %, and 0.35 %, respectively. Liquid phase spectrogram of these three different fingerprinting methods was shown in **Figure S1**, **Figure S2**, and **Figure S3**, respectively.

**Table S2** Percentage contents results of Ginsenoside Rg1, Ginsenoside Re, and Ginsenoside Rb1.

| Method | Ginsenoside Rg1 (%) | Ginsenoside Re (%) | Ginsenoside Rb1  (%) |
| --- | --- | --- | --- |
| Pharmacopoeia method | 1.33 ± 0.01 | 0.25 ± 0.01 | 0.36 ± 0.01 |
| Methanol method | 1.19 ± 0.02 | 0.23 ± 0.00 | 0.33 ± 0.00 |
| 70 % Ethanol method | 1.24 ± 0.01 | 0.24 ± 0.00 | 0.35 ± 0.01 |


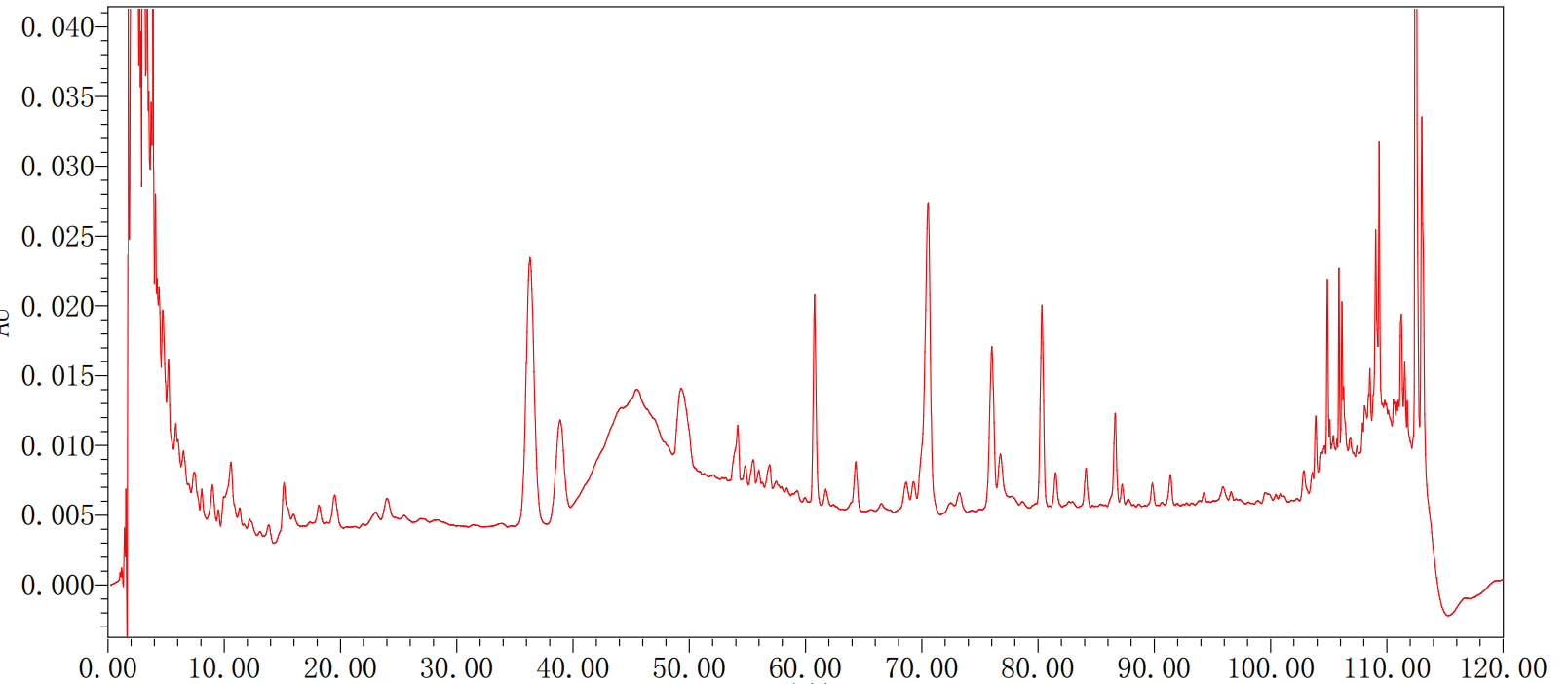


Ginsenoside Re

Ginsenoside Rg1

Ginsenoside Rb1

**Figure S1** Liquid phase spectrogram of pharmacopoeia method.


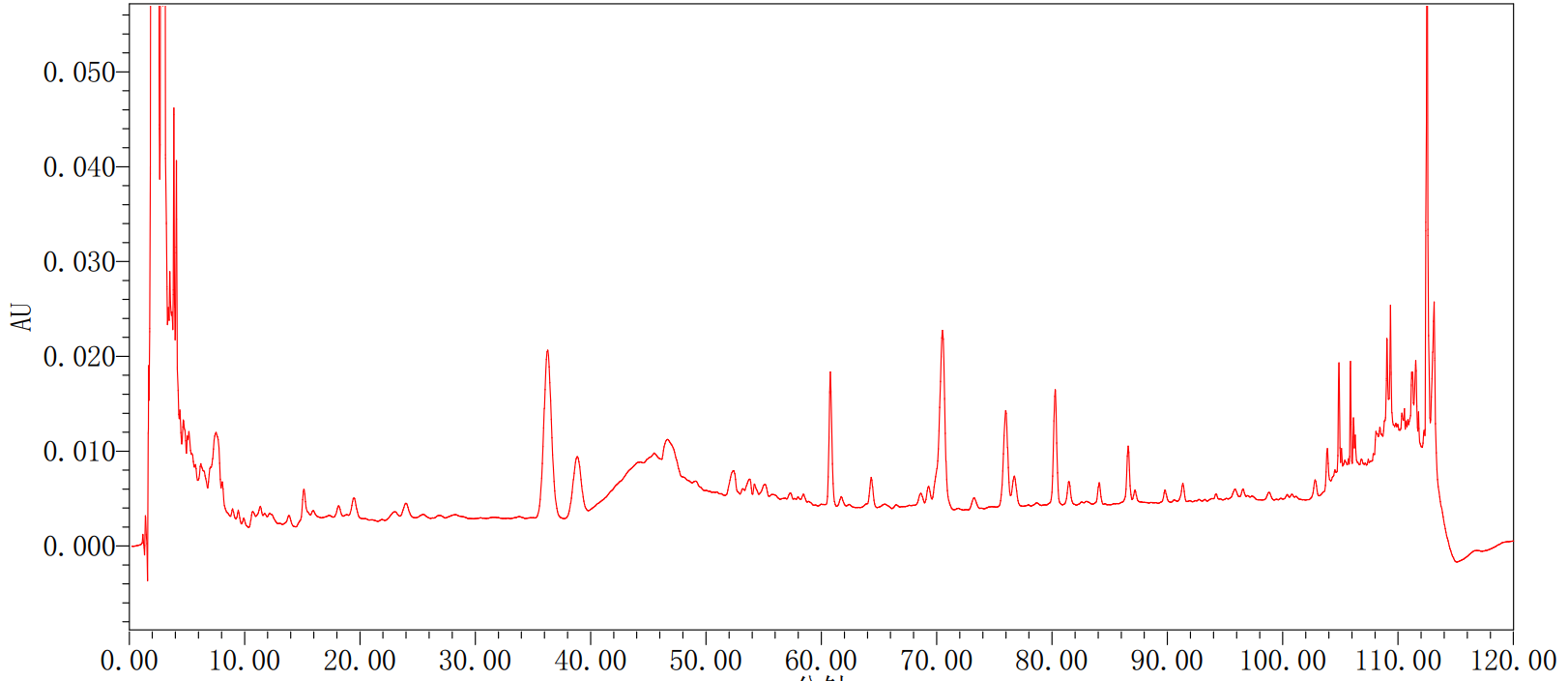


Ginsenoside Re

Ginsenoside Rg1

Ginsenoside Rb1

**Figure S2** Liquid phase spectrogram of methanol method.


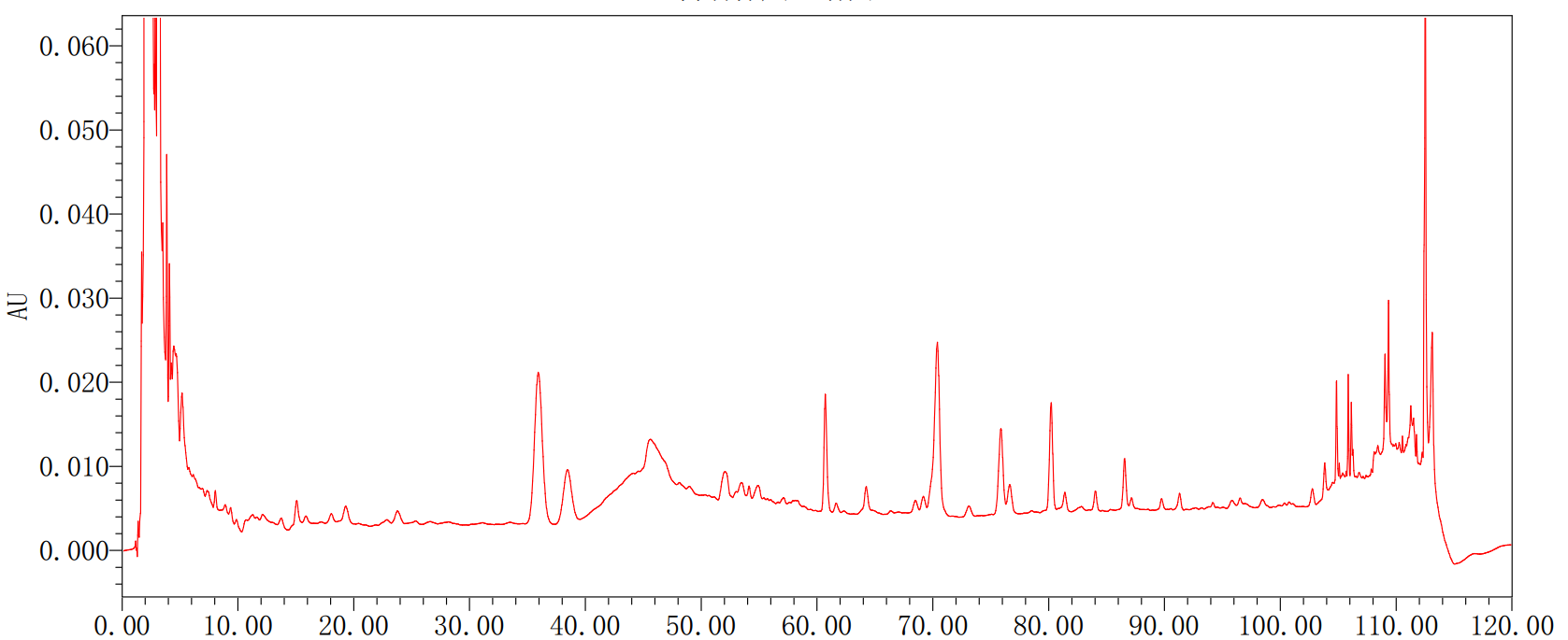


Ginsenoside Re

Ginsenoside Rg1

Ginsenoside Rb1

**Figure S3** Liquid phase spectrogram of 70 % ethanol method.
